# Non-contiguous finished genome sequence and description of *Kurthia massiliensis* sp. nov.

**DOI:** 10.4056/sigs.3206554

**Published:** 2012-12-14

**Authors:** Véronique Roux, Khalid El karkouri, Jean-Christophe Lagier, Catherine Robert, Didier Raoult

**Affiliations:** 1 Aix Marseille Université, URMITE, Faculté de médecine, Aix-Marseille Université

**Keywords:** *Kurthia massiliensis*, *Firmicutes*, capsule, Flagella

## Abstract

*Kurthia massiliensis* strain JC30^T^ sp. nov. is the type strain of *K. massiliensis* sp. nov., a new species within the genus *Kurthia*. This strain, whose genome is described here, was isolated from the fecal flora of a healthy patient. *K. massiliensis* is a Gram-positive aerobic rod. Here we describe the features of this organism, together with the complete genome sequence and annotation. The 3,199,090 bp long genome contains 3,240 protein-coding genes and 86 RNA genes, including between 3 and 4 rRNA genes.

## Introduction

*Kurthia massiliensis* strain JC30^T^ (CSUR 141^T^ = DSM 24639^T^) is the type strain of *K. massiliensis* sp. nov. This bacterium is a Gram-positive, strictly aerobic rod that is capsulated, and motile by peritrichous flagella. This organism was originally isolated from the stool of a healthy Senegalese patient as part of a "culturomics" study aimed at cultivating all species within human feces, individually.

Currently, "the gold standard” for defining bacterial species is DNA-DNA hybridization [1]. But this method is time-consuming and the inter-laboratory reproducibility is poor. Fortunately, the development of PCR and next-generation sequencing technologies have led to reliable and reproducible 16S rRNA comparison methods with generally agreed upon cutoff values that enable the taxonomic classification of new species for many bacterial genera [2]. To describe new bacterial taxa, the use of a polyphasic approach was proposed [3] that includes their genome sequence, MALDI-TOF spectrum and main phenotypic characteristics (habitat, Gram-stain reaction, cultivation conditions, cell wall structure and metabolic characteristics).

The genus *Kurthia* was created in 1885 by Trevisan [4] in honor of Kurth who described the first species, *Bacterium zopfii*, isolated from the intestinal contents of chickens. As the stool samples had been stored at room temperature and the bacteria were strictly aerobic, it was assumed that the samples were contaminated by *Kurthia*, which multiplied during storage. The name *Kurthia* was first published in the seventh edition of *Bergey’s Manual of Determinative Bacteriology* [5] and was included in the Approved Lists of Bacterial Names [6]. Currently, Kurthia includes 3 species: *K. zopfii*, *K. gibsonii* [7] and *K. sibirica* [8]. The bacteria are members of the phylum *Firmicutes*, and the family *Planococcaceae*. There is no evidence of pathogenicity.

Here we present a summary classification and a set of features for *K. massiliensis* sp. nov. strain JC30^T^ together with the description of the complete sequencing and annotation of its genome. These characteristics support the circumscription of the species *K. massiliensis*.

## Classification and features

**Figure 1:**
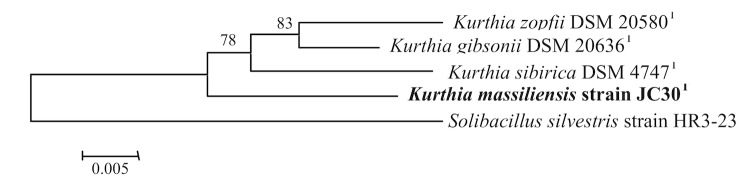
Phylogenetic tree highlighting the position of *Kurthia massiliensis* strain JC30^T^ relative to other type strains within the genus *Kurthia*. Sequences were aligned using CLUSTALX, and phylogenetic inferences obtained using the neighbor-joining method within the MEGA 5 package [20]. Numbers at the nodes are percentages of bootstrap values obtained by repeating the analysis 1,000 times to generate a majority consensus tree. *Solibacillus silvestris* was used as outgroup. The scale bar represents 0.005 nucleotide change per nucleotide position.

A stool sample was collected from a healthy 16-year-old male Senegalese volunteer patient living in Dielmo (a rural village in the Guinean-Sudanian zone in Senegal), who was included in a research protocol. The patient gave an informed and signed consent, and the agreement of the National Ethics Committee of Senegal and the local ethics committee of the IFR48 (Marseille, France) were obtained under agreement 09-022. The fecal specimen was preserved at -80°C after collection and sent to Marseille. Strain JC30 (Table 1) was isolated in January 2011 by aerobic cultivation on 5% sheep blood-enriched Columbia agar (BioMerieux). This strain exhibited a 96.9% nucleotide sequence similarity with *K. gibsonii*, the phylogenetically closest validated *Kurthia* species (Figure 1). This value was lower than the 97% 16S rRNA gene sequence threshold to delineate a new species without carrying out DNA-DNA hybridization recommended by the report of the *ad hoc* committee on reconciliation of approaches to bacterial systematics [2]. Stackebrandt and Ebers proposed to increase this value to 98.7% [21].

**Table 1:** Classification and general features of *Kurthia massiliensis* strain JC30^T^ according to the MIGS recommendations [9]

**MIGS ID**	**Property**	**Term**	**Evidence code^a^**
	Current classification	Domain *Bacteria*	TAS [10]
		Phylum *Firmicutes*	TAS [11,12]
		Class *Bacilli*	TAS [13,14]
		Order *Bacillales*	TAS [6,15]
		Family *Planococcaceae*	TAS [6,16]
		Genus *Kurthia*	TAS [4,6,17,18]
		Species *Kurthia massiliensis*	IDA
		Type strain JC30^T^	IDA
	Gram stain	Positive	IDA
	Cell shape	Coccobacilli	IDA
	Motility	Motile by peritrichous flagella	IDA
	Sporulation	Nonsporulating	IDA
	Temperature range	Mesophile	IDA
	Optimum temperature	37°C	IDA
MIGS-6.3	Salinity	Growth in BHI medium + 3% NaCl	IDA
MIGS-22	Oxygen requirement	Aerobic	IDA
	Carbon source	Unknown	NAS
	Energy source	Unknown	NAS
MIGS-6	Habitat	Human gut	IDA
MIGS-15	Biotic relationship	Free living	IDA
MIGS-14	Pathogenicity Biosafety level Isolation	Unknown 2 Human feces	NAS
MIGS-4	Geographic location	Senegal	IDA
MIGS-5	Sample collection time	September 2010	IDA
MIGS-4.1	Latitude	13.7167	IDA
MIGS-4.1	Longitude	16.4167	IDA
MIGS-4.3	Depth	Surface	IDA
MIGS-4.4	Altitude	51 m above sea level	IDA

Evidence codes - IDA: Inferred from Direct Assay; TAS: Traceable Author Statement (i.e., a direct report exists in the literature); NAS: Non-traceable Author Statement (i.e., not directly observed for the living, isolated sample, but based on a generally accepted property for the species, or anecdotal evidence). These evidence codes are from the Gene Ontology project [19]. If the evidence is IDA, then the property was directly observed for a live isolate by one of the authors or an expert mentioned in the acknowledgements.

**Figure 2:**
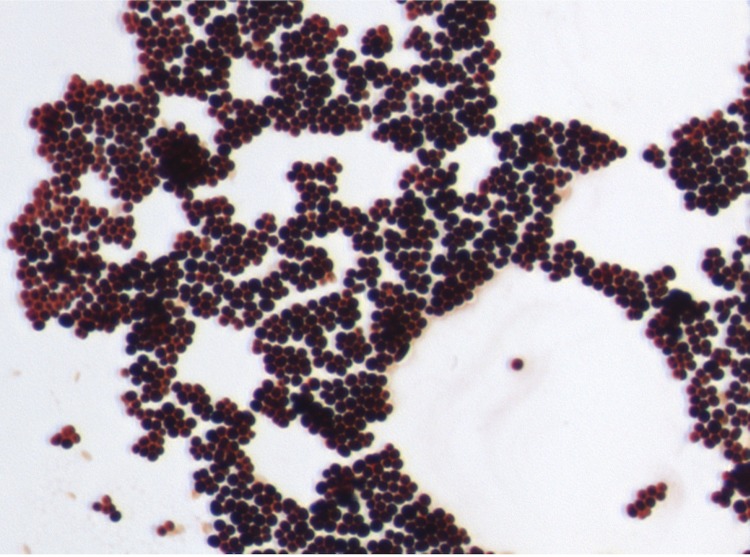
Gram staining of *K. massiliensis* strain JC30^T^

Surface colonies were observed on sheep blood agar (bioMérieux) after 24 h aerobic incubation at 37°C. The colonies of strain JC30^T^ were circular, greyish/yellowish, shiny, curved and smooth, 2-5 mm in diameter. Gram staining showed Gram-positive coccobacilli (Figure 2).

Different growth temperatures (25, 30, 37, 45, 50 and 55°C) were tested. Growth occurred between 25°C and 55°C, and optimal growth was observed between 25°C and 50°C. Growth of the strain was tested under aerobic atmosphere, in the presence of 5% CO_2_, and under anaerobic and microaerophilic atmospheres, which were created using GENbag anaer and GENbag microaer (bioMérieux), respectively. The strains were aerobic but also grew under microaerophilic conditions and in the presence of 5% CO_2_. Growth does not occur under anaerobic conditions. NaCl tolerance of strain JC30^T^ was determined on Difco^TM^Brain Heart Infusion Agar plates (Becton Dickinson). The powder was supplemented with NaCl (Euromedex) to obtain the tested concentrations (0.5, 1, 2, 3, 5 10, 15%, w/v). Growth occurred between 0.5-5% NaCl but the optimum growth was between 0.5-3% NaCl. Growth in the range of pH 5.0-10.0 was tested using BBL^TM^ Brain Heart Infusion (Becton Dickinson). pH tolerance revealed that growth could occur over a range of pH 6.0 – 9.0 with optimal growth between pH 7.0 - 9.0.

**Figure 3:**
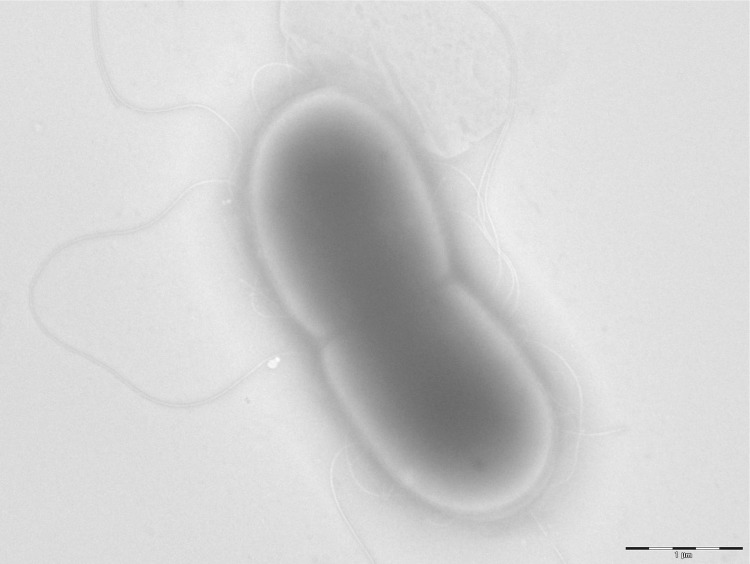
Transmission electron microscopy of *K. massiliensis* strain JC30^T^, using a Morgani 268D (Philips) at an operating voltage of 60kV.The scale bar represents 1 μm.

**Figure 4:**
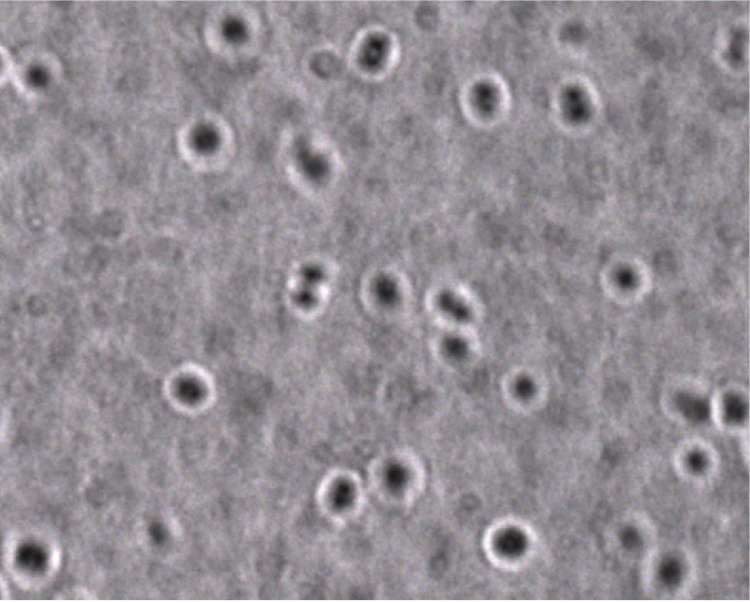
India ink capsule stain of *K. massiliensis*

**Figure 5:**
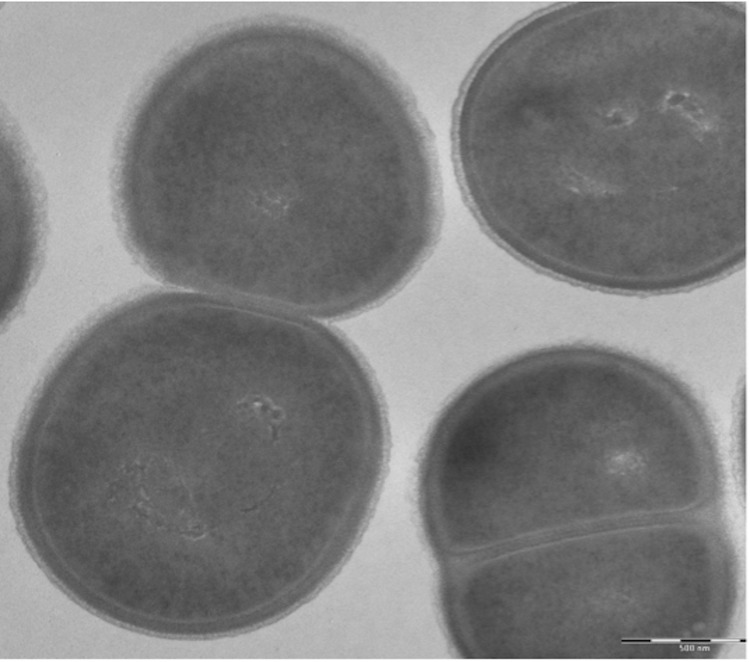
Capsule characterization of *K. massiliensis* after the bacteria were embedded in Epon 812 resin and observed by transmission electron microscopy.

The size and ultrastructure of cells were determined by negative staining transmission electron microscopy. The rods were 0.9-2.4 μm long and 0.6-1.8 μm wide (Figure 3). Peritrichous flagella were observed. Capsule presence was determined by India ink stain and after bacteria embedding in Epon 812 resin and observation by transmission electron microscopy (Figures 4 and 5). Strain JC30^T^ exhibited catalase activity but no oxidase activity. Api ZYM, Api 20NE (BioMérieux) were used to study biochemical characters [Table 2].

**Table 2:** Differential phenotypic characteristics between strain JC30^T^ and related species

Characteristic	1	2	3	4
gelatin hydrolysis	+	-	-	-
N-acetyl-glucosamine assimilation	-	-	+	-
D-maltose assimilation	+	-	-	-
potassium gluconate assimilation	+	-	-	-
trisodium citrate assimilation	+	-	-	-
alkaline phosphatase	-	+	w	+
esterase (C4)	+	+	w	w
esterase lipase (C8)	+	+	w	w
valine arylaminidase	w	-	+	-
cystine arylaminidase	+	+	-	-
α-chemotrypsin	w	-	+	-
naphtol-AS-BI-phosphohydrolase	-	-	+	+
α-glucosidase	+	-	-	-

Strains: 1, *K. massiliensis* sp. nov. JC30^T^; 2, *K. gibsonii* DSM 20636^T^; 3, *K. zopfii* DSM 20580^T^; 4, *K. sibirica* DSM 4747^T^.

+: positive result, -: negative result, w: weak positive result

Analysis of respiratory quinones by HPLC was carried out by the Identification Service and Dr Brian Tindall, DSMZ, Braunschweig, Germany. Respiratory lipoquinones were extracted from 100 mg of freeze dried cell material as described by Tindall [22,23]. Respiratory lipoquinones were separated into their different classes (menaquinones and ubiquinones) by thin layer chromatography on silica gel, using hexane:*ter*-butylmethylether (9:1 v/v) as solvent. UV absorbing bands corresponding to menaquinones or ubiquinones were removed from the plate and further analyzed by HPLC with detection at 269 nm. The only respiratory quinone for strain JC30^T^ was MK-7 (100%). Preparation and determination of cellular fatty acids were carried out by following the procedures given for the Sherlock Microbial identification System (MIDI). The major fatty acids were C_15:0_ iso 68.04% and C_15:0_ anteiso 16.92%. Polar lipids were extracted from 100 mg of freeze dried cell material using a chloroform:methanol:0.3% aqueous NaCl mixture 1:2:0.8 (v/v/v) (modified after [24]). The extraction solvent was stirred overnight and the cell debris pelleted by centrifugation. Polar lipids were recovered into the chloroform phase by adjusting the chloroform:methanol:0.3% aqueous NaCl mixture to a ratio of 1:1:0.9 (v/v/v). Polar lipids were separated as previously described [25]. The polar lipids present were diphosphatidylglycerol, phosphatidylglycerol, phosphatidylethanolamine, phospholipid 1. The peptidoglycan of strain JC30^T^ was isolated as described by Schleifer [26]. Analysis was carried out as previously described [26,27] with the modification that TLC on cellulose was used rather than paper chromatography. Quantitative analysis of amino acids was performed following derivatization by gas chromatography and gas chromatography / mass spectrometry (320-MS Quadrupole GC/MS, Varian) [28]. *K. massiliensis* showed the peptidoglycan type A4αL-Lys←D-Glu (type A11.33 according to reference [36] ).

*K. massiliensis* was susceptible to penicillin G, amoxicillin, amoxicillin + clavulanic acid, imipenem, gentamycin, erythromycin, doxycycline, rifampicin, vancomycin, and nitrofurantoin. The organism was resistant to ceftriaxone, ciprofloxacin, sulfamethoxazole trimethoprim and metronidazole.

**Figure 6:**
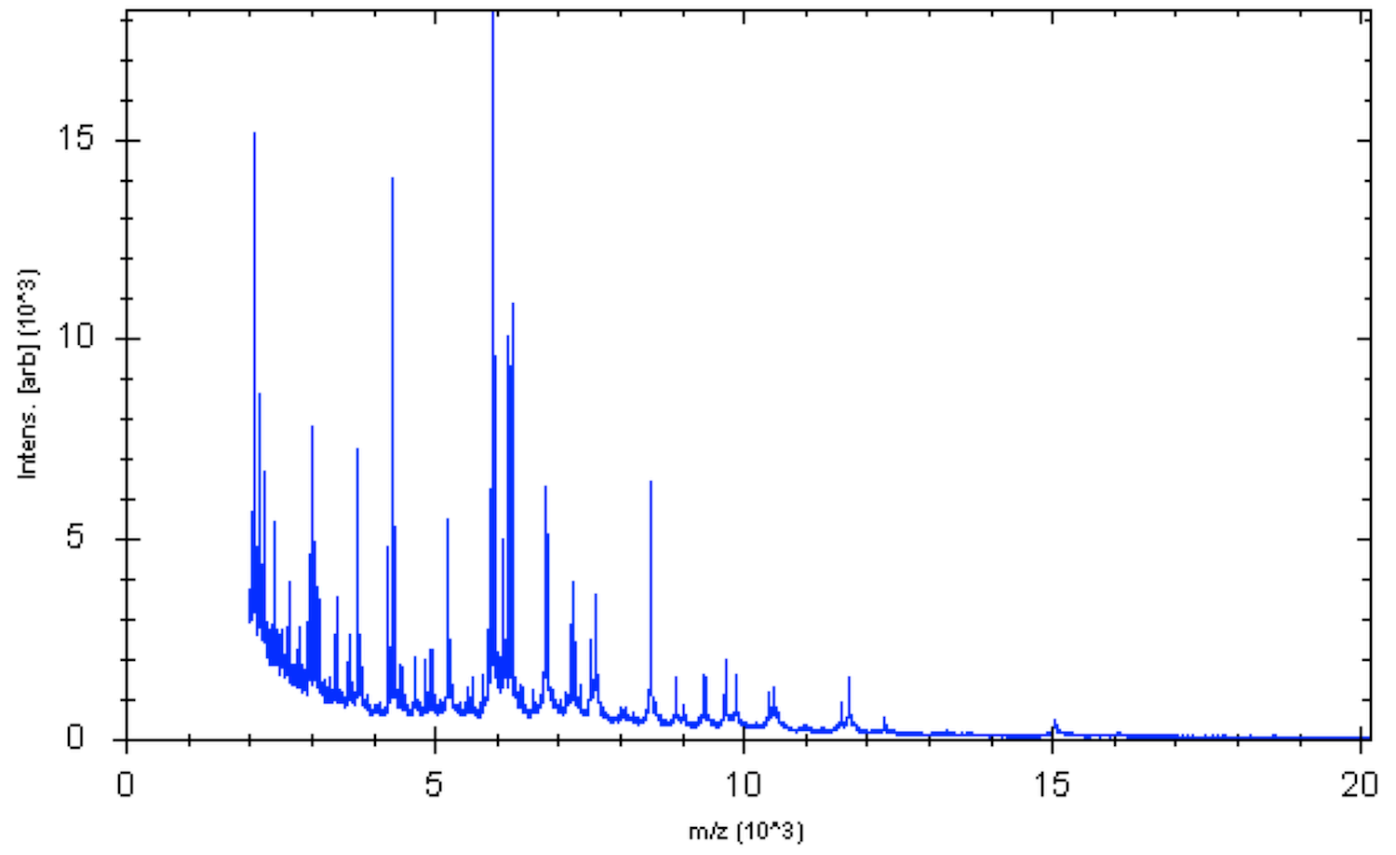
Reference mass spectrum from *K. massiliensis* strain JC30^T^. Spectra from 24 individual colonies were compared and a reference spectrum was generated.

Matrix-assisted laser-desorption/ionization time-of-flight (MALDI-TOF) MS protein analysis was carried out. Briefly, a pipette tip was used to pick one isolated bacterial colony from a culture agar plate, and to spread it as a thin film on a MALDI-TOF target plate (Bruker Daltonics). Twelve distinct deposits were made for strain JC30^T^ from twelve isolated colonies and the manipulation was repeated another day. After air-drying, 1.5 µl matrix solution (saturated solution of α-cyanohydroxycinnaminic acid in 50% aqueous acetonitrile containing 2.5% trifluoroacetic acid) per spot was applied. MALDI-TOF MS was conducted using the Microflex LT spectrometer (Bruker Daltonics). All spectra were recorded in linear, positive ion mode. The acceleration voltage was 20 kV. Spectra were collected as a sum of 240 shots across a spot. Preprocessing and identification steps were performed using the manufacturer’s parameters. The JC30^T^ spectra were imported into the MALDI BioTyper software (version 3.0, Bruker) and analyzed by standard pattern matching (with default parameter settings) against the main spectra of 4,108 bacteria including those from *K. gibsonii*, K. sibirica** and *K. zopfii*, used as reference data, in the BioTyper database. A score enabled the identification, or not, from the tested species: a score > 2.3 with a validly published species enabled the identification at the species level, a score > 1.7 but < 2 enabled the identification at the genus level; and a score < 1.7 did not enable any identification. For strain JC30^T^, none of the obtained scores was > 1, thus suggesting that our isolate was not a member of a known species. We incremented our database with the spectrum from strain JC30^T^ (Figure 6). The spectrum was made available online in our free-access URMS database [29].

## Genome sequencing information

### Genome project history

The organism was selected for sequencing on the basis of its phylogenetic position and 16S rRNA similarity to other members of the genus *Kurthia*, and is part of a “culturomics” study of the human digestive flora aiming at isolating all bacterial species within human feces. It was the first genome of a *Kurthia* species A summary of the project information is shown in Table 3. The EMBL accession number is CAEU01000000 and consists of 98 contigs (≥200 bp) and 18 scaffold (> 2,424 bp). Table 3 shows the project information and its association with MIGS version 2.0 identifiers.

**Table 3:** Project information

**MIGS ID**	**Property**	**Term**
MIGS-31	Finishing quality	High-quality draft
MIGS-28	Libraries used	One paired end 3-kb library and one Shotgun library
MIGS-29	Sequencing platforms	454 GS FLX Titanium
MIGS-31.2	Fold coverage	22×
MIGS-30	Assemblers	Newbler version 2.5.3
MIGS-32	Gene calling method	Prodigal
	EMBL ID	CAEU01000000
	EMBL Date of Release	February 12, 2012
	Project relevance	Study of the human gut microbiome

### Growth conditions and DNA isolation

*K. massiliensis* sp. nov. strain JC30^T^, CSUR P141^T^, DSM 24639^T^, was grown aerobically on 5% sheep blood-enriched Columbia agar at 37°C. Three petri dishes were spread and resuspended in 3×100 µl of G2 buffer. A first mechanical lysis was performed by glass powder on the Fastprep-24 device (Sample Preparation system) from MP Biomedicals, USA using 2×20 second cycles. DNA was then treated with lysozyme (4.17g/L, 30 minutes at 37°C) and extracted through the BioRobot EZ 1 Advanced XL (Qiagen). The DNA was then concentrated and purified on a Qiamp kit (Qiagen). The yield and the concentration were measured by the Quant-it Picogreen kit (Invitrogen) on the Genios Tecan fluorometer at 63.1/µl.

### Genome sequencing and assembly

Shotgun and 3-kb paired-end sequencing strategies were used. The shotgun library was constructed with 500 ng of DNA with the GS Rapid library Prep kit (Roche). For paired-end sequencing, 5 µg of DNA was mechanically fragmented on a Hydroshear device (Digilab) with an enrichment size at 3-4 kb. The DNA fragmentation was visualized using the 2100 BioAnalyzer (Agilent) on a DNA labchip 7500 with an optimal size of 3.619 kb. The library was constructed according to the 454 GS FLX Titanium paired-end protocol. Circularization and nebulization were performed and generated a pattern with an optimal size of 472 bp. After PCR amplification through 15 cycles followed by double size selection, the single stranded paired-end library was then quantified using the Genios fluorometer (Tecan) at 430 pg/µL. The library concentration equivalence was calculated as 1.69E+09 molecules/µL. The library was stored at -20°C until further use.

The shotgun and paired-end libraries were clonally-amplified with 3 cpb and 1cpb in 3 and 4 emPCR reactions respectively on the GS Titanium SV emPCR Kit (Lib-L) v2 (Roche). The yields of the emPCR were 18.65 and 14.31% respectively. Approximately 340,000 beads for the shotgun sequencing and 790,000 beads for the 3kb paired end sequencing were loaded onto the GS Titanium PicoTiterPlate PTP Kit 70×75 and sequenced with the GS FLX Titanium Sequencing Kit XLR70 (Roche). The run was performed overnight and then analyzed on the cluster through the gsRunBrowser and Newbler assembler (Roche). A total of 294,263 passed filter wells were obtained and generated 81.3 Mb with a length average of 301 bp. The passed filter sequences were assembled using Newbler with 90% identity and 40 bp as overlap. The final assembly identified 18 scaffolds and 72 large contigs (>1,500 bp).

### Genome annotation

Coding sequences (CDSs) were predicted using PRODIGAL with default parameters [30]. The functional annotation of protein sequences was performed against the non-redundant GenBank database using BLASTP. Functional categories of these proteins were searched against the Clusters of Orthologous Groups (COG) database using COGNITOR [31]. The prediction of RNAs genes, i.e., rRNAs, tRNAs and other RNAs was carried out using RNAmmer [32] and ARAGORN [33] algorithms. The transmembrane segments and peptide signals were identified using TMHMM [34] and SignalP tools [35].

## Genome properties

**Figure 7:**
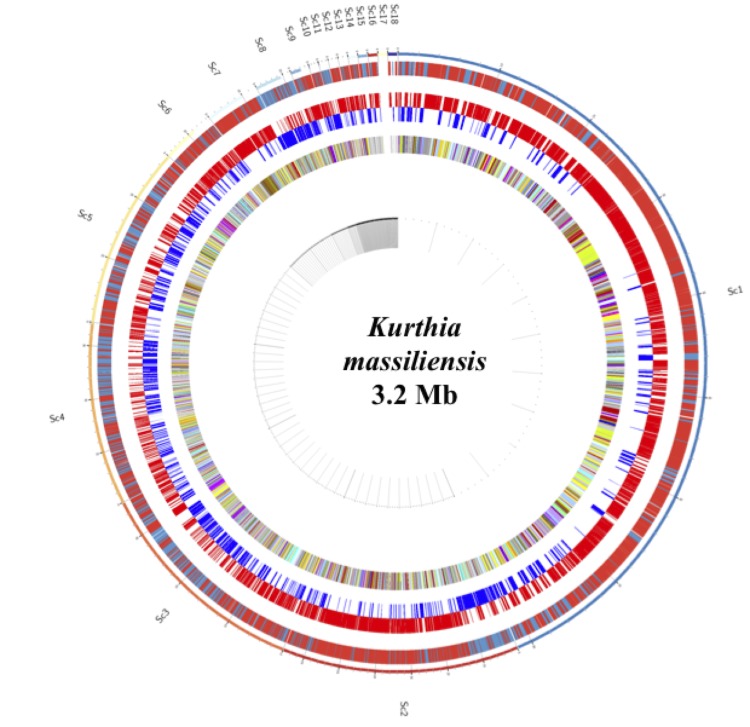
Graphical circular map of *Kurthia massiliensis* genome. From outside to the center: Genes on both strands, genes on foward strand, genes on reverse strand and genes colored by COG categories.

The genome is 3,199,090 bp long with a 39.26% GC content (Table 4, Figure 7). Of the 3,326 predicted genes, 3,240 were protein-coding genes, and 86 were RNAs. A total of 2,425 genes (74.8%) were assigned a putative function. The remaining genes were annotated as either hypothetical proteins or proteins of unknown functions. The distribution of genes into COGs functional categories is presented in Table 5. The properties and the statistics of the genome are summarized in Tables 4 and 5.

**Table 4:** Nucleotide content and gene count levels of the genome

Attribute	Value	% of total^a^
Genome size (bp)	3,199,090	
DNA coding region (bp)	2,794,828	87.4
DNA G+C content (bp)	1,255,894	39.26
Total genes	3,326	100
RNA genes	86	2.6
Protein-coding genes	3,240	97.4
Genes with function prediction	2,425	74.8
Genes assigned to COGs	2,500	77.16
Genes with peptide signals	269	8.3
Genes with transmembrane helices	473	14.6

a) The total is based on either the size of the genome in base pairs or the total number of protein coding genes in the annotated genome.

**Table 5:** Number of genes associated with the 25 general COG functional categories

**Code**	**Value**	**%age**	**Description**
J	161	4.97	Translation
A	0	0	RNA processing and modification
K	218	6.73	Transcription
L	184	5.68	Replication, recombination and repair
B	1	0.03	Chromatin structure and dynamics
D	34	1.05	Cell cycle control, mitosis and meiosis
Y	0	0	Nuclear structure
V	47	1.45	Defense mechanisms
T	171	5.28	Signal transduction mechanisms
M	118	3.64	Cell wall/membrane biogenesis
N	82	2.53	Cell motility
Z	0	0	Cytoskeleton
W	0	0	Extracellular structures
U	43	1.33	Intracellular trafficking and secretion
O	92	2.84	Posttranslational modification, protein turnover, chaperones
C	139	4.29	Energy production and conversion
G	134	4.14	Carbohydrate transport and metabolism
E	267	8.24	Amino acid transport and metabolism
F	74	2.28	Nucleotide transport and metabolism
H	134	4.14	Coenzyme transport and metabolism
I	110	3.40	Lipid transport and metabolism
P	204	6.30	Inorganic ion transport and metabolism
Q	68	2.10	Secondary metabolites biosynthesis, transport and catabolism
R	402	12.41	General function prediction only
S	237	7.31	Function unknown
X	740	22.84	Not in COGs

The total is based on the total number of protein coding genes in the annotated genome.

## Comparison with other *Kurthia* genomes

To date, no genome of other strains or species belonging to the genus *Kurthia* were sequenced.

## Conclusion

On the basis of phenotypic, phylogenetic and genomic analyses, we formally propose the creation of *Kurthia massiliensis* sp. nov., which contains the strain JC30^T^. This bacterium was found in Senegal.

## Description of *Kurthia massiliensis* sp. nov.

*Kurthia massiliensis* (mas.si.li.en'sis. L. masc. adj. *massiliensis* of Massilia, the old Roman name for Marseille, where the type strain was isolated). Isolated from stool of a healthy Senegalese patient. *K massiliensis* are aerobic Gram-positive coccobacilli. On sheep blood agar after 24 h aerobic incubation at 37°C, colonies of strain JC30^T^ are circular, greyish/yellowish, shiny, curved and smooth, 2-5 mm in diameter. Cells are motile by peritrichous flagella and encapsulated. Catalase activity is positive but oxidase activity is negative. Gelatine hydrolysis, maltose assimilation, potassium gluconate assimilation, malic acid assimilation, trisodium citrate assimilation are present. Esterase (C4), esterase lipase (C8), cystine arylaminidase, α-gluconidase activities are observed. Valine arylaminidase and alpha-chemotrypsin activities are weakly positive. The major fatty acids are *iso* C_15:0_ 68.04% and *anteiso* C15:0 16.92%. Polar lipids found are diphosphatidylglycerol, phosphatidylglycerol, phosphatidylethanolamine, and phospholipid 1. The peptidoglycan type is A4αL-Lys←D-Glu (type A11.33 according to [36]). Cells are susceptible to penicillin G, amoxicillin, amoxicillin + clavulanic acid, imipenem, gentamycin, erythromycin, doxycycline, rifampicin, vancomycin and nitrofurantoin. The G+C content of the genome is 39.26%. The type strain is JC30^T^ (= CSUR P141^T^ = DSM 24639^T^).
